# Technical and Economic Evaluation of WWTP Renovation Based on Applying Ultrafiltration Membrane

**DOI:** 10.3390/membranes10080180

**Published:** 2020-08-07

**Authors:** He Bai, Yakai Lin, Hongbin Qu, Jinglong Zhang, Xiaohong Zheng, Yuanhui Tang

**Affiliations:** 1 Beijing Scinor Membrane Technology Co. Ltd., Bejing 100083, China; bai.he@scinormem.com (H.B.); qu.hongbin@scinormem.com (H.Q.); zhang.jinglong@scinormem.com (J.Z.); zheng.xiaohong@scinormem.com (X.Z.); 2 Beijing Key Laboratory of Membrane Materials and Engineering, Department of Chemical Engineering, Tsinghua University, Beijing 100084, China; 3 College of Chemistry and Environmental Engineering, China University of Mining and Technology, Beijing 100083, China

**Keywords:** WWTP, renovation and upgrading, ultrafiltration membrane, net present value

## Abstract

Nowadays, the standards of discharging are gradually becoming stricter, since much attention has been paid to the protection of natural water resources around the world. Therefore, it is urgent to upgrade the existing wastewater treatment plant (WWTP), to improve the effluent quality, and reduce the discharged pollutants to the natural environment. In this paper, taking the “Liaocheng UESH (UE Envirotech) WWTP in Shandong province of China” as an example, the existing problems and the detailed measures for a renovation were systemically discussed by technical and economic evaluation, before and after the renovation. During the renovation, the ultrafiltration membrane was added as the final stage of the designed process route, while upgrading the operation conditions of biochemical process at the same time. After the renovation, the removal rates of chemical oxygen demand (COD_cr_), biochemical oxygen demand (BOD_5_), total phosphorus (TP) and other major pollutants were improved greatly, and the results fully achieved the standards of surface water class IV. The ultrafiltration system performs a stable permeability around 1.5 LMH/kPa. Besides, the economic performance of the renovation was evaluated via the net present value (NPV) method. The result reveals that the NPV of the renovation of the WWTP within the 20 year life cycle is CNY 72.51 million and the overall investment cost can be recovered within the fourth year after the reoperation of the plant. This research does not only indicate that it is feasible to take an ultrafiltration membrane as the main technology, both from technical and economic perspectives, while upgrading the biochemical process section in the meantime, but also provides a new strategy for the renovation of existing WWTPs to achieve more stringent emission standards.

## 1. Introduction

Pollution to natural water resource is a worldwide emergent and critical problem. It is definite that the severe deterioration of water bodies has both short- and long-term negative effects to human and environmental health [1]. In particular, chemicals and microbial contaminants in treated wastewater would cause public health concerns [2]. In recent years, wastewater discharging has gained increasing attention in many countries, due to reasons of ensuring water security and developing effective strategies for the sustainable utilization of water resources. Some developed countries have formulated different policies or standards on the discharge of wastewater treatment plant (WWTP) [3]. The point source emission standard of the United States, which has experienced a shift in policy direction from “technologically based” to “water bodies-based” in recent years, requires the limit of water discharged to sensitive water body of total nitrogen (TN) < 3 mg/L, total phosphorus (TP) < 0.1 mg/L [3,4]. The Japanese government also proposed a special emission standard limits for Osaka Bay of BOD_5_ < 8 mg/L, TN < 8 mg/L and TP < 0.8 mg/L.

Developing countries are facing a more serious deterioration situation of natural water bodies with the development of their economy and the improvement of industrialization. Many countries have taken steps to limit the emission of pollutants [5]. Taking China as an example, only chemical oxygen demand (COD_cr_), biochemical oxygen demand (BOD_5_), suspended solids (SS) and other major pollutants were taken into consideration for the emission standards in the early years. Later, the nutrient salts, nitrogen and phosphorus were added in the emission standard list that needed to be controlled as conventional indicators. Currently, the Chinese government has further restricted nutrient emissions and many local governments have issued even stricter local emission standards according to the natural environment in different regions [6]. Hence, it is needed to develop simple, reasonable and acceptable treatment strategies for the renovation of existing wastewater plants in response to increasingly stringent emission standards. It will reduce the discharge of pollutants into natural water bodies; and eliminate negative impacts on the environment and human health, which would be in accordance with the national and international water quality regulations and guidelines.

Since normally urban wastewaters are only treated by conventional activated sludge systems without further treatment [7], the effluents quality can no longer meet the requirements of current emission standards, especially the indicators of SS and nutritive salts due to a lack of technical process as well as equipment [6,7]. Because of its potential economic and environmental benefits, the renovation of existing WWTP to fix water deterioration is regarded as one of the best options for protecting natural water bodies and developing sustainable water management strategies. However, the renovation is a difficult decision for many plant managers, because the renovation of a WWTP is directly related to the total construction costs, operating costs, treatment effect, the floor area, the convenience of management and other key issues. The selection of the technology and the full use of existing facilities are important for the renovation. If only simply modifying the existing system but not adding new technology, no remarkable success would be achieved in significantly decreasing the concentrations of major pollutants, to the levels stated in the restricted criteria corresponding to the water discharged to the natural environment. However, there are numerous problems, such as the footprint, time limit and the difficulty in estimating the expected benefit. Moreover, it is the fate that the WWTP effluents flow to densely populated urban cities paradoxically in order to protect the local natural environment. Therefore, there is a growing need for the development of treatment renovation methodologies by considering the cost-effective and technical benefits.

Today, the implementation of ultrafiltration membrane in wastewater reclamation and reuse has become more attractive, since ultrafiltration membrane separation ensures a higher removal rate of particles, bacteria and large molecular weight organic matters as well as reducing chemical usage and better on-stream time [8,9]. However, there are few applications incorporating the biochemical system for the pretreatment that could keep the inlet water quality of ultrafiltration membrane stable. In addition, there is also a lack of systematic experience in the selection of ultrafiltration membrane products. Therefore, although ultrafiltration membrane is receiving more and more attention in recent years, however, some people worry that the investment is too large to be recovered, or about the rapid contamination of the membrane, and others do not know how to systematically evaluate the renovation methodologies. The study of Al Aani et al. shows that fouling (27%), modelling (17%) and wastewater reuse (12%) were the dominant research topics for the ultrafiltration membrane, however, there is very little research on ultrafiltration membrane for the renovation of existing WWTP [10]. Therefore, it results in a lack of evidence for the WWTP manager to follow about the ultrafiltration membrane and the upgrading of pretreatment, either in a technical or an economic aspect. [11]. Moreover, the incomplete or insufficient economic analyses of options by ultrafiltration membrane processes for wastewater discharge do not allow to balance or accurately evaluate the disparity among the benefits brought by the increase in water price after the renovation and overall investment cost of the whole plant renovation [12].

Considering the current situation summarized above, taking the Liaocheng UESH WWTP in Shandong province of China as an example, this work introduces a renovation process route with ultrafiltration as the main technology and analyzes the existing problems and the specific measures for the renovation. Then, based on continuously monitoring the operation data of ultrafiltration performances, the actual renovation effects and economic feasibilities of membrane treatments were studied using the net present value method. By way of technical and economic perspective, the viability of the renovation methodologies that take ultrafiltration as the main technology and combines with the upgrading of conventional activated sludge systems in the production of discharged water from urban WWTP effluents was elaborately evaluated, in order to provide a new direction for the renovation of existing WWTP to accommodate more stringent emission standards.

## 2. Background

The UESH WWTP is located in the Liaocheng Economic Development Zone (Liaocheng, China) and it was put into operation in May 2009 with a designed capacity of 30,000 m^3^/d. The plant mainly treats the domestic sewage in the economic development zone and a part of industrial sewage from enterprises. The conventional method of A/A/O (Anaerobic-Anoxic-Oxic) technology with steps in anaerobic, anoxic and aerobic was adopted as the main part of the treatment process, supplemented by biological phosphorus removal methods to achieve the purpose of nitrogen and phosphorus removal. The effluent quality met the primary-level A-class standard of “Pollutant Discharge Standards of Urban Sewage Treatment Plants” (GB 18918-2002) that is shown in Table 1, and then the treated wastewater could be discharged into the Haihe River (Liaocheng, China), the local natural water body.

However, with an increase in effort from the Chinese government for the protection of natural water bodies, various policies have been formulated and promulgated, and the discharge standards implemented by the sewage plant could no longer meet the needs of protecting the local natural water environment. Both the “Action Plan for Water Pollution Prevention and Control” that was formulated and promulgated by the State Council in 2015 and the “Work Plan of Liaocheng City Water Pollution Prevention and Control in 2017” that was formulated and issued by the Liaocheng People’s Government clearly require that the water quality of the Haihe River, to which the effluent is discharged from the plant, should reach the Class IV of “Quality Standard of Surface Water” [13], which is shown in Table 2. Therefore, it was urgent for the plant to carry out some renovation in order to reduce the pollution load and protect the local natural environment.

### 2.1. Plant Current Condition

**Figure 1:**
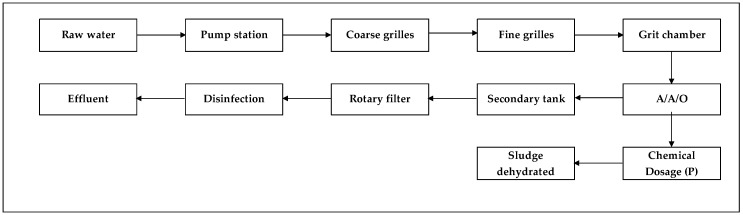
Treatment process of the plant before renovation.

The plant is running at a full capacity of 30,000 m^3^/d, and the flow diagram of the treatment process is shown in Figure 1. The sewage was pre-treated by a grilles and grit chamber, and then treated by A/A/O conventionally activated sludge method. The effluent from the secondary sedimentation tank was treated by a rotary filter, and then discharged into the receiving water body after disinfection. The concentrated and dehydrated sludge would be transported to a company specializing in sludge disposal for further treatment. The main pollutant indicators of the influent and effluent from the Liaocheng UESH WWTP before the renovation are shown in Table 3.

As listed in Table 3, the variation range of organic matter content was large, the BOD_5_ of the influent was around 40–80 mg/L, while the COD_Cr_ fluctuated between 130 mg/L and 250 mg/L, and the suspended solid was about 100 mg/L. The concentration of nutrient salts was relatively stable, ammonia nitrogen was at about 15 mg/L, and the total phosphorus fluctuated around 3 mg/L. Besides, according to the description of a field operator, the sludge concentration in the aerobic tank was relatively high, with an MLSS (Mixed Liquor Suspended Solid) value at 8000–11,000 mg/L and MLVSS (Mixed Liquor Volatile Suspended Solid) at 3400–4400 mg/L, and the MLVSS was only about 40% and the sludge load was only 0.02 kg BOD5/(MLVSS.d), which signified the poor sludge sedimentation ability of the previous treatment process. Therefore, the biochemical properties of the water also needed to be improved.

In addition, due to the long-term full capacity or even overload operation of the plant, there also existed a serious loss of facilities, equipment aging and other problems, such as the bad performance of the aeration distribution system that was caused by the corrosion of the pipelines, which resulted in the uneven mixing of sludge and water in the aerobic tank and an unsatisfactory flow state. The failure of the propeller affected the uneven mixing of sludge and water even further. Besides, the rotary filter was easily fouled and the inefficient backwash made the SS of the effluent unstable.

As shown in Table 3, although the effluent quality could meet the primary-level A-class standard of “Pollutant Discharge Standards of Urban Sewage Treatment Plants”, however, except for the ammonia nitrogen, the concentration of other main pollutant indicators, especially the total phosphorus and suspended solid, were still much higher than the requirement of the Class IV standard listed in Table 2. In order to improve the effluent quality of the plant, it was necessary to carry out the plant renovation as soon as possible, to reduce the concentration of the main indicators and gradually restore the regional water ecological function.

### 2.2. Selection of Renovation Methodologies

The new standard requires the concentration of total nitrogen (TN), total phosphorus (TP) and suspended solid (SS) of the effluent to be much lower. The removal of dissolved substances still needs to be treated by biochemical process, while the removal of suspended solid needed to be further treated. The effluent SS was about 10 mg/L, and it was difficult to achieve the corresponding standards of 6 mg/L only by the rotary filter due to its easily fouling rate and inefficient backwash. Considering the possible high SS that was caused by chemical dosage in the biochemical process after the renovation, a new treatment process needed to be added in order to keep the effluent quality stable. The newly added treatment process should reach the characteristics of small footprint and compact structure because of the limitation of land area, with only 720 m^2^ available, which became one of the major difficulties for the renovation. In addition, features such as the high degree of automation and stable effluent water quality also needed to be taken into consideration. The optional processes included the ultrafiltration membrane, sand filtration combined with ozone and other processes, while the ultrafiltration has obvious advantages due to the operational safety and land saving.

The membrane filtration system is a pressure-driven separation process, in which particles and impurities between 0.02 and 0.1 μm in diameter can be intercepted through the micro pores distributed on the membrane surface, which can effectively remove water floc, bacteria and macromolecular organic matter [14]. The ultrafiltration membrane among the relatively mature technologies from recent years, especially after entering the 21st century, which has rapidly developed into a utility engineering technology, which is widely used in various fields of water treatment and become more competitive compared with traditional technologies, because of the scale production of membrane materials, the integration of membrane modules, the popularization of membrane manufacture and the affordable prices [15]. Not only all bacteria and suspended solids are trapped by the efficient intercept of the ultrafiltration membrane, but also the COD_cr_, the total phosphorus and total nitrogen carried by a suspended substance, which realized the further protection of effluent quality after biochemical treatment. Additionally, some refractory macromolecular organic matter can be retained and returned to the biochemical tank by backwashing, in order to prolong its residence time and maximize its degradation.

After a certain period of operation of the ultrafiltration membrane, the retained pollutants will be accumulated on the membrane surface and formed into a filter cake layer that would reduce the membrane flux [14,15,16]. Therefore, it is necessary to maintain good inlet water quality to protect the stable operation of the ultrafiltration membrane, prolong the cleaning cycle and increase its service life. As a result, the pretreatment facilities also needed to be upgraded.

Through the analysis of the main pollutant of inlet water and production requirements, the effluent COD_cr_ was about 37 mg/L, so it is necessary to maintain a good biochemical performance, to prevent the membrane from fouling, and at the same time, the ultrafiltration membrane could help to further reduce the concentration of COD_cr_. Besides, the high concentration of ammonia nitrogen (NH_3_–N) and the total nitrogen (TN), as well as the low carbon source in the raw water, were not conducive to denitrification and could affect the performance of phosphorus removal at the same time. Moreover, there was a risk of effluent short circuit on the existing complete mixing of activated sludge that would easily result in sludge swelling, which can also influence the performance of denitrification and phosphorus removal. How to improve the efficiency of denitrification and phosphorus removal in the A/A/O process was one of the technical difficulties for the upgrading of biochemical treatment.

The influent BOD_5_/NH_3_–N was about 3.5 while the NH_3_–N/COD_cr_ was about 0.09, showing a lack of carbon source in the biochemical treatment process, which previously resulted in the relatively unstable level of effluent TN content [17]. In addition, as the ratio of BOD_5_ to TP was about 20, which meant that the biological phosphorus removal process can be adopted [18], but in order to improve the removal rate of total phosphorus, so as to meet the stricter phosphorus removal target, not only was there a need to optimize the biochemical treatment process, but also to carry out an auxiliary chemical phosphorus removal system.

**Figure 2:**
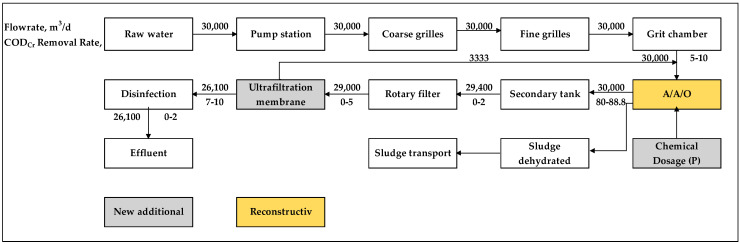
Treatment process of the plant before renovation.

The renovation was required to tap the potential of the existing facilities, and new technology needed to be added at the same time to make the effluent water quality fully up to the standard. Furthermore, reducing the investment and operation cost as much as possible, as well as achieving the convenient operation and management of the WWTP should also be taken into consideration. Therefore, the final renovation methodology of the plant was determined to be the upgrading of the existing biochemical treatment process, the addition of a chemical phosphorus removal system and an ultrafiltration membrane treatment process. The treatment process after renovation is shown in Figure 2.

### 2.3. Implementation of Renovation

#### 2.3.1. Upgrading of A/A/O Treatment Process

Considering the existing problems of uneven mixing, short flow and serious sludge accumulation at the bottom of the biochemical tank, which resulted in the unstable performance of the effluent quality, following specific renovation measures should be carried out while keeping the original structure. Giving the returned sludge inlet the same position as the feed water inlet in the anaerobic tank, and optimizing the arrangement of mixer at the same time, allows the water and sludge to fully mix with the kinetic energy during the water feed, meanwhile, it also enables to make full use of the anaerobic tank volume during the mixture. The anoxic tank has the same problems as the anaerobic tank. Similarly, by adjusting the position of the returned sludge inlet port and optimizing the arrangement of mixer, the mixed logistics state of the sludge and water in the anoxic tank can be improved, so as to improve the treatment efficiency of the anoxic zone.

In order to solve the problem of insufficient carbon source in the influent, a carbon source feeding device is set near the anoxic tank to periodically and quantitatively transport a proper amount of high concentration and high biodegradability sewage and sodium acetate, after accounting as a supplementary carbon source.

There used to be a short flow problem for the design of internal reflux, which was that the water could flow from the inlet port of the aerobic tank directly into the anaerobic tank. Therefore, a separation wall was added to block the short flow. In addition, the aeration in the reflux water from the aerobic tank to the anaerobic tank is greatly reduced, to avoid affecting the performance of the anaerobic zone.

#### 2.3.2. Upgrading of Phosphorus Removal System

The original biochemical phosphorus removal process is still used. To increase the phosphorus removal rate, chemical agents are added to make phosphorus form into insoluble substances, so as to be discharged together with the residual sludge [19]. Hence, a new chemical phosphorus removal and dosing device was redesigned in the renovation. The new liquid phosphorus removal agent, whose main component is ferric sulfate, can be used continuously and automatically.

#### 2.3.3. Addition of Ultrafiltration Membrane

The ultrafiltration system includes the ultrafiltration membrane and membrane frame, inlet water pump, backwash system, chemical cleaning system, pipe valve, compressed air system, instrument and automatic control system, among which the core part is the ultrafiltration membrane. The SMT600 series of the pressurized ultrafiltration membrane were selected for the plant renovation. PVDF hollow fiber ultrafiltration membrane is generally produced by thermally induced phase separation (TIPS) process or non-solvent induced phase separation process (NIPS). Compared with NIPS membranes, TIPS membranes, which are prepared driven by a temperature change, have many advantages such as an easily controllable structure, stable membrane quality, narrow micro pore distribution and symmetric structure [20,21]. Moreover, the characteristics of PVDF raw materials can be maintained during manufacturing, which makes the membranes possess higher mechanical strength and chemical resistance. Therefore, the ultrafiltration membranes fabricated by the TIPS method can tolerate a high concentration of soaking and cleaning. Moreover, the advantages of the TIPS membrane can minimize the number of membrane modules due to higher flux and good recoverability, so as to save the floor space and prolong the service life [21]. Table 4 describes the technical parameters of the ultrafiltration membranes used in this renovation.

## 3. Evaluation Method

### 3.1. Technical Analysis

The purpose of the renovation of the Liaocheng UESH WWTP was to improve the removal rate of the main treatment indicators in the water to achieve a level Class IV of “Water Quality Standard of Surface Water”, so as to protect the receiving natural water body. Both the old standard and the new standard did not make clear provisions on calcium, magnesium and carbonate content for the wastewater that discharged into natural water bodies, as new standards mainly made more stringent requirements on COD, nitrogen, phosphorus and other nutritive salts. Moreover, since there is no reverse osmosis after the UF (Ultrafiltration) process, thus the scaling problem does not need to be taken into consideration, hence, the concentration of these ions is not measured by the plant. As the traditional municipal sewage, the content of these ions is not high, which is acceptable for the operation of the UF membrane. Therefore, this paper focuses on the concentration of the main pollutant in the effluent and the change of the removal rate of before and after the renovation as a part of the technical evaluation.

On the other hand, ultrafiltration membrane permeability will be used to investigate the effect of biochemical process upgrading and the stability of the whole process after renovation. One of the characteristics of ultrafiltration membrane fouling is the increase in transmembrane pressure difference (TMP) and the drop of permeability [22]. This paper focuses on the changes of the membrane permeability, which is usually expressed as the flowrate per hour per square meter of membrane area under unit pressure. The influence of pretreatment on the membrane fouling rate as well as the cleaning effect and recovery performance can be evaluated by the continuous monitoring of membrane permeability.

### 3.2. Economical Analysis

The net present value method of the dynamic evaluation index in engineering economics is used for economic evaluation in this paper. The economic evaluation index is divided into static and dynamic, where static evaluation means that the time value of the fund will be not taken into account and the compound interest will be not calculated when calculating the benefits and costs of the scheme, while they will be taken into account by the dynamic evaluation, of which the calculation process is based on the equivalent basic conversion formula, which includes the net present value [23].

The total cost of the system in its whole life cycle is the sum of the construction, operation, maintenance and energy costs. However, since the changes in the time value of money, the project costs occurring at different points in the asset life cycle cannot be compared or simply added together. They must be discounted to their present value. Appropriate formula for the net present value is as follows [24]:

(1) $$\mathrm{NPV}=\sum \frac{\left(C_{I}-C_{O}\right)}{{\left(1+i\right)}^{t}}$$

where, NPV = net present value; *C_I_* = cash inflows; *C_0_* = cash outflows; *i* = discount rate in decimals; *t* = time period.

The result of the NPV method is more realistic because it takes the time value of money into account and it also considers the risk inherent in making projections about the future. Hence this method is useful in the rational arrangement and financial management of the future costs and activities of the WWTP.

## 4. Results and Discussion

### 4.1. Technical Results

**Figure 3:**
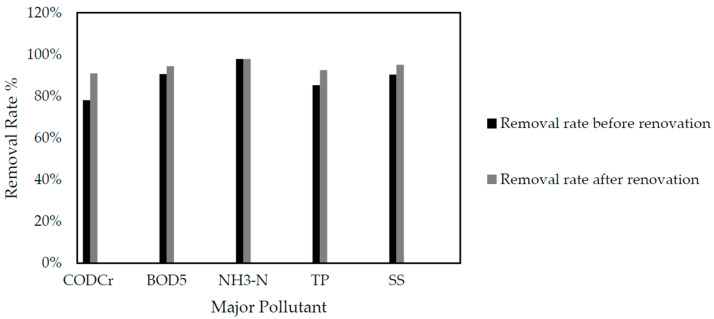
Removal rate of the major pollutants before and after the plant renovation, as well as the comparison of effluent water quality with the new discharge standards.

Table 5 shows the concentration of a major pollutant in the influent and effluent as well as the removal rate after the plant renovation. Figure 3 shows the improvement of removal rate of major pollutants before and after the plant renovation. The data were collected from the semi-annual water quality analysis report of the plant in 2019.

It can be seen from Figure 3, that the removal rates of COD_cr_, BOD_5_, TN, TP and SS were significantly improved after the renovation, while maintaining the removal rate of ammonia nitrogen in the original level. The concentrations of major pollutants in the effluent are much lower, which totally meet the requirement of the new discharge standard. Among them, the water and sludge can be more fully mixed due to the adjustment of the water inlet port and the sludge inlet port as well as the improvement of the thruster, and the hydraulics flow pattern of the reflux between each biochemical tank is also improved, so as to avoid the formation of dead sludge accumulated in the tank [25]. The removal rate of BOD_5_ is increased to 94% after the renovation, which is better than 91% of that in the original process. In addition, with the interception of a certain amount of macromolecular organic matter by the ultrafiltration membrane, the COD_cr_ removal rate was greatly increased from 78% to 91%.

Chemical agents also have a positive effect on the removal of total phosphorus. Usually the biological phosphorus removal method cannot achieve the ideal effect because it is very sensitive to temperature, water salinity and other aspects. After the additional chemical phosphorus removal method, the phosphorus is changed into insoluble phosphate precipitation form by adding iron salt phosphorus removal agent. On the one hand, iron combines with phosphoric acid, and on the other hand, its hydrolysates can form Fe(OH)_3_ and other complexes, which make the original colloids in the water destabilized by adsorption bridging and net capture and sweep, so as to be flocculated and precipitated, which is much easier after it is combined into macromolecules [26]. Compared with the biological phosphorus removal method only, the total phosphorus removal rate was increased from 85% to 93% by adding the chemical phosphorus removal system.

**Figure 4:**
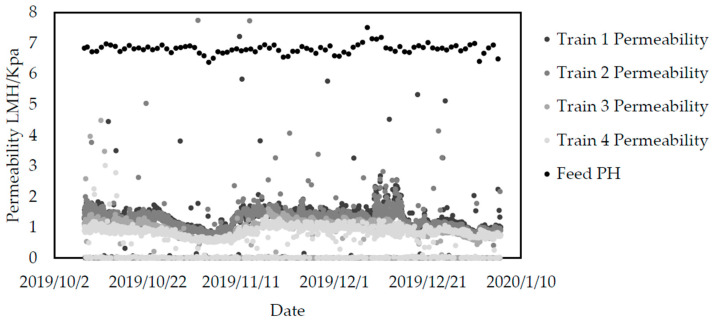
Permeability trend of the ultrafiltration membrane within three months after the renovation.

The ultrafiltration membranes, which are the key technology of the renovation, have a significant interception rate for suspended solid, and then the concentration of SS in the effluent is almost impossible to be detected, which is far lower than the required 6 mg/L. Meanwhile, the upgrading of the biochemical treatment also benefits the operation performance of the ultrafiltration membrane. Figure 4 describes the permeability trend of the ultrafiltration membrane within three months after the renovation.

The newly added ultrafiltration membrane was divided into four sets, each set with 80 membrane modules, each of which could operate independently. As shown in Figure 4, the ultrafiltration membrane permeability of the four sets remains basically stable in the operation for three consecutive months. In late October to early November of 2019, there was an impact dosage of the chemical phosphor removal agent, which resulted in the rapid fouling of the membrane and decline of the permeability [27]. However, it was returned to the initial level after one time of chemical cleaning, which certified a good recoverable performance of the membranes. The permeability of the membrane system was basically maintained at about 1.5 LMH/kPa, which was in the higher level compared with the other ultrafiltration membrane on the market [28].

### 4.2. Economical Results

The NPV method was used to evaluate the economy performance of the renovation. The cash inflow is the sewage treatment fee charged by the plant after the renovation, while the cash outflow includes the initial investment cost, operation and maintenance cost [29]. The cost of operation and maintenance mainly include the cost of phosphorus removal agent, the cost of carbon source supplement and the ultrafiltration membrane cleaning agent, power consumption and labor cost of the new equipment, while the data are collected from the plant. The depreciation cost of the ultrafiltration membrane was calculated based on the warranty period given by the membrane manufacturer. The details are as follows:

The total investment cost of this renovation was CNY 25.626 million.

The power consumption mainly included the consumption of ultrafiltration feed pump, backwash pump, metering pump and other power equipment, as well as the power consumption of the newly added lighting and control device. It was calculated that the additional power consumption of the project was 2.199 million KWH per year.

The chemical consumption mainly comes from the chemical phosphor removal, the supplemented carbon source and the cleaning of the ultrafiltration membrane. The main agents that were newly added were sodium acetate, ferric sulfate new agents, sodium hypochlorite, hydrochloric acid and sodium hydroxide. According to the design value, the main chemical consumption was shown in Table 6. According to the three-month operation, the actual consumption was lower than the design value. However, considering uncertain factors such as water quality fluctuation in the future, the design value will be used for the calculation of economic evaluation.

The depreciation cost of the ultrafiltration membrane was calculated according to the six year warranty period given by the manufacturer, and the local electricity price, labor cost and pharmaceutical price were calculated according to the local average price of the last two years. The annual maintenance cost was calculated at 2% of the investment cost.

The unit sewage treatment fee charged by the water plant was CNY 2.76/ton after the renovation, and the water treatment fee per ton was CNY 1.58 higher than the original CNY 1.18 before the renovation. Thus, the CNY 1.58/ton will be used as the calculation basis for cash the inflow during the economic evaluation.

**Figure 5:**
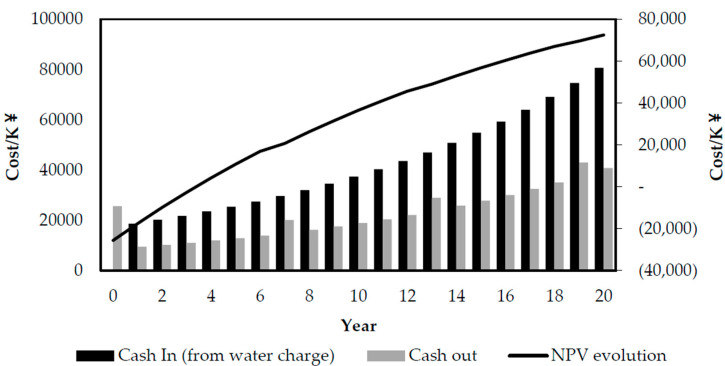
Net present value (NPV) calculation result.

In addition, the base year for the NPV calculation WAs 2019, which WAs the commissioning stage after the renovation. The NPV analysis requires a discount rate calculated using interest rates and inflation rates [30]. Interest rates and inflation are based on the historical data of the past 25 years. The average interest rate is calculated as 14% and the average inflation rate is 8% after the average value is collected, hence, the calculated discount rate is 5.55%. All capital inflows and outflows were converted into the present value of the base year in 2019, and then added by the NPV method to obtain the cost of different calculation life cycle. All the calculations were completed using MS Office Excel, and the results are shown in Figure 5.

Due to the improvement of water quality after the renovation, the cost of sewage treatment fee was greatly increased. As shown in Figure 5, the net present value within the 20 year life cycle is CNY 72.51 million, and the overall cost can be recovered in the fourth year after the renovation, which brings considerable economic benefits to the plant.

**Figure 6:**
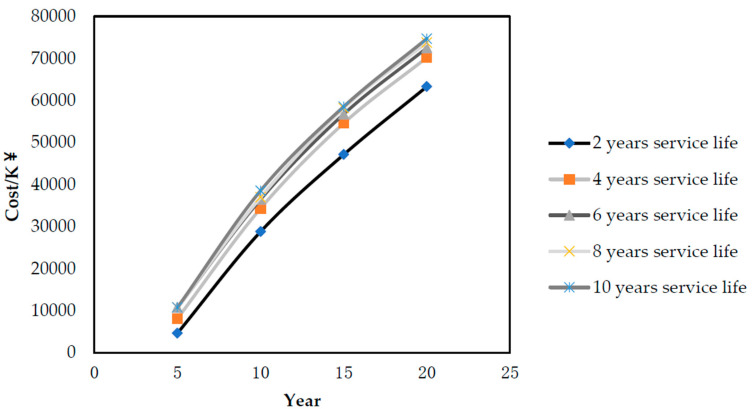
NPV calculation results under the different membrane life cycles.

In addition, it can be seen that one of the variables of this renovation methodology was the selection of ultrafiltration membrane. There are different kinds of ultrafiltration membranes with different materials, different performance, operation stabilities and life cycles on the market, which directly affect the economic benefits of water plants by the chemical consumption, membrane depreciation cost and other factors [31]. This paper takes the service life of the ultrafiltration membrane as an example to study the influence of different membrane replacement cycles on the NPV result, as shown in Figure 6.

Figure 6 shows the NPV result of the water plant from 5 to 20 years with the different ultrafiltration membrane service life cycles. It can be seen that different ultrafiltration membranes replacement cycles have a direct impact on the NPV value of the whole life cycle of the plant. When the service life of the membrane is less than 3 years, the influence is much more significant. Ultrafiltration membrane species also affect chemical consumption, power consumption and other factors, and then the selection of ultrafiltration membrane is important in the renovation based on the process route introduced in this paper. The TIPS ultrafiltration membrane was used in the Liaocheng USEH WWTP, which could benefit the water plant continuously due to its good chemical resistance and recoverability.

### 4.3. Challenges and Future Research Orientations

It is obvious that the ultrafiltration membrane will play an increasingly important role in wastewater treatment plant renovation [10]. However, while the ultrafiltration membrane technology is constantly innovating, the research on the mechanism of membrane fouling by specific pollutants is insufficient, moreover, the market supervision is also deficient, which is manifested in the following aspects. Firstly, there is a lack of an integrated outline and systematic standard for ultrafiltration membrane evaluation in the technical and economic dimensions; in addition, there is also no widely accepted operation standards, which result in a lack of evidence for the WWTP to follow during management [10,32,33]. Therefore, it can be predicted that the future research orientation will be more inclined to build the evaluation system of ultrafiltration membrane, and to determine the weight of each indicator, in order to propose a comprehensive and systematic outline of technical and economic dimensions. Additionally, membrane fouling, shrinkage, cycles of operation, and regeneration prospects also need be further studied in the future. In a wastewater treatment process, the physically irreversible fouling of ultrafiltration membranes is severe and inevitable, the permeability loss restricts the application of ultrafiltration for wastewater treatment, and it will also reduce the service life of the membrane and increase the cost of membrane replacement [32,33,34]. The key issue to solve the fouling problem is to understand the fouling mechanism and cleaning efficiency of a specific pollutant, and find an effective way to regenerate the membrane [35,36,37]. In addition, how to select and upgrade pretreatments, which can represent important savings in the operational costs related to the membrane’s cleaning procedures and maintenance, will also be one of the future research orientations [38].

However, due to the limitation of the site spaces and time, no other membrane products were performed in this project. Therefore, by reading a large number of studies, the author compared the application of different ultrafiltration membranes in other projects, especially in a municipal wastewater field, and summarized the relevant factors affecting the performance of the ultrafiltration membrane [39]. Meanwhile, we proposed an integrated outline for the evaluation of ultrafiltration membrane-based renovation methodologies of the technical and economic dimensions, which are presented based on the actual Chinese market situation, as shown in Table 7 and Table 8 [40,41], which can be regarded as a reference for the establishment of ultrafiltration membrane evaluation systems in the future.

## 5. Conclusions

In this work, taking the Liaocheng UESH WWTP as an example, this research proves that from the technical perspective, it is a feasible scheme to take the ultrafiltration membrane as the main technology and upgrade the biochemical process section in the meantime. Due to the high efficiency of the ultrafiltration membrane interception characteristics, the main pollutant in the effluent after the renovation could totally meet the Class IV requirement of “Water Quality Standard of Surface Water”. At the same time, the upgrading of the biochemical treatment can also reduce the fouling rate of the ultrafiltration membrane and keep a stable operation status, and thus bring a beneficial impact on the local natural environment. Economic performances evaluated by the NPV method have clearly demonstrated that based on the operational perspective, the ultrafiltration membrane represents a highly competitive technological solution. Thus, we anticipate that the ultrafiltration membrane would play an important role in the renovation of WWTPs. Meanwhile, systematic evaluation systems and research on the fouling mechanism of the ultrafiltration membrane will be the emphases of future research and development.

## Figures and Tables

**Table 1:** Pollutant discharge standards of urban sewage treatment plants.

Nr.	Items	Primary-Level	
		A-Class	B-Class
1	Chemical oxygen demand (COD_Cr_)	50 mg/L	60 mg/L
2	Biochemical oxygen demand (BOD_5_)	10 mg/L	20 mg/L
3	Suspended solids (SS)	10 mg/L	20 mg/L
4	Animal and plant oil	1 mg/L	3 mg/L
5	Petroleum	1 mg/L	3 mg/L
6	Anionic surfactant	0.5 mg/L	1 mg/L
7	Total nitrogen (in N)	15 mg/L	20 mg/L
8	Ammonia nitrogen (in N)	5 (8) mg/L	8 (15) mg/L
9	Total phosphorus (in P)	0.5 mg/L	1 mg/L
10	Chroma (dilution)	30 mg/L	30 mg/L
11	pH	6–9	6–9
12	Number of fecal coliforms (1/L)	103	104

**Table 2:** Water quality standards of surface water.

Nr.	Items	Class I	Class II	Class III	Class IV	Class V
1	Temperature °C	Weekly mean maximum temperature rise ≤ 1 Weekly mean maximum temperature drop ≤ 2				
2	pH	6–9				
3	Dissolved oxygen (DO) ≥ mg/L	7.5	6	5	3	2
4	Chemical oxygen demand (COD_Cr_) mg/L	15	15	20	30	40
5	Biochemical oxygen demand (BOD_5_) mg/L	3	3	4	6	10
6	Total nitrogen (in N) (TN) mg/L	0.2	0.5	1.0	1.5	2.0
7	Ammonia nitrogen (in N) (NH_3_–N) mg/L	0.15	0.5	1.0	1.5	2.0
8	Total phosphorus (in P) (TP) mg/L	0.02	0.1	0.2	0.3	0.4

**Table 3:** Main pollutant indicators of the influent and effluent from the wastewater treatment plant (WWTP) before renovation *.

Months		1	2	3	4	5	6	7	Average
COD_Cr_ (mg/L)	Influent	202	180	237	166	142	142	138	78%
	Effluent	41	47	38	30	28	35	40	
	Removal rate	80%	74%	84%	82%	80%	75%	71%	
BOD_5_ (mg/L)	Influent	67.2	61.6	78.4	52.5	56.8	45.2	59.4	91%
	Effluent	5.9	6.4	5.6	5.1	5	4.8	6.2	
	Removal rate	91%	90%	93%	90%	91%	89%	90%	
NH_3_–N (mg/L)	Influent	15.97	14.87	16.47	15.42	17.01	16.14	14.1	98%
	Effluent	0.25	1.21	0.22	0.15	0.26	0.17	0.14	
	Removal rate	98%	92%	99%	99%	98%	99%	99%	
TP (mg/L)	Influent	3.24	3.3	2.71	2.46	2.45	2.43	2.85	85%
	Effluent	0.64	0.74	0.13	0.19	0.35	0.34	0.58	
	Removal rate	80%	78%	95%	92%	86%	86%	80%	
SS (mg/L)	Influent	102	105	146	113	93	117	97	90%
	Effluent	10	10	11	9	9	13	12	
	Removal rate	90%	90%	92%	92%	90%	89%	88%	

* The data came from the semi-annual water report of the plant in 2017.

**Table 4:** Main pollutant indicators of the influent and the effluent from WWTP before renovation.

Items	Parameters
Model	SMT600-P80
Material	PVDF
Pore size (μm)	0.1
Membrane area (m^2^)	80
Nominal size (mm)	Φ225 * 2360
Resistance to NaClO (ppm)	5000

**Table 5:** Main pollutant indicators of the influent and effluent from the WWTP after renovation.

Months		10	11	12	Average	Standard
COD_Cr_ (mg/L)	Influent	171	202	164	91%	30 mg/L
	Effluent	18	17	15		
	Removal rate	89%	92%	91%		
NH_3_–N (mg/L)	Influent	20.3	28.19	27	99.8%	1.5 mg/L
	Effluent	0.05	0.05	0.07		
	Removal rate	99.7%	99.8%	99.7%		
TP (mg/L)	Influent	2.31	3.24	2.42	93%	0.3 mg/L
	Effluent	0.17	0.16	0.16		
	Removal rate	93%	95%	93%		
SS (mg/L)	Influent	72	95	83	98%	5 mg/L
	Effluent	1.65	1.43	1.84		
	Removal rate	98%	98%	98%		

**Table 6:** Additional chemical consumption after renovation.

Nr.	Chemical Agents	Dosage Quantity (kg/d)
1	Sodium hypochlorite	76
2	Sodium hydroxide	14.6
3	Hydrochloric acid	12.78
4	Phosphorus removal agent	6075.4
5	Sodium acetate	8000

**Table 7:** Technique indexes of the ultrafiltration membrane system-based WWTP renovation methodology evaluation.

Evaluation Item		Weight
Renovation methodologies	Effluent quality	++++
	Automaticity	++
	Security	++
	Installation convenience	++
	Floor space	+
	Construction difficulty	++
	Stability	++
Ultrafiltration membrane	Chemical tolerance	+++
	Material safety	++
	Warranty period	+++
	Cleaning period	+
	Fouling resistant	++
	Integrity	+++
	Environmental adaptability	+

**Table 8:** Economic indexes of the ultrafiltration membrane system-based WWTP renovation methodology evaluation.

Evaluation Item		Weight
Total investment costs	Including equipment, materials, construction, installation	+++
Operating costs	Including power consumption costs, chemical consumption, costs, labor costs and management fees	+++
Sewage treatment fee		+++
